# The Asia Pacific Disaster Mental Health Network: Setting a Mental Health Agenda for the Region

**DOI:** 10.3390/ijerph17176144

**Published:** 2020-08-24

**Authors:** Elizabeth A. Newnham, Peta L. Dzidic, Enrique L.P. Mergelsberg, Bhushan Guragain, Emily Ying Yang Chan, Yoshiharu Kim, Jennifer Leaning, Ryoma Kayano, Michael Wright, Lalindra Kaththiriarachchi, Hiroshi Kato, Tomoko Osawa, Lisa Gibbs

**Affiliations:** 1 School of Psychology, Curtin University, Perth 6845, Australia; peta.dzidic@curtin.edu.au (P.L.D.); enrique.mergelsberg1@curtin.edu.au (E.L.P.M.); 2 François-Xavier Bagnoud Center for Health & Human Rights, Harvard University, Boston, MA 02115, USA; emily.chan@cuhk.edu.hk (E.Y.Y.C.); jleaning@hsph.harvard.edu (J.L.); 3 Centre for Victims of Torture, Kathmandu 44600, Nepal; bhushan@cvict.org.np; 4 Division of Global Health and Humanitarian Medicine, CUHK, Hong Kong 999077, China; 5 Nuffield Department of Medicine, University of Oxford, Oxford OX3-7LF, UK; 6 National Institute of Mental Health, Tokyo 187-0031, Japan; joice21@yc4.so-net.ne.jp; 7 World Health Organization Kobe Centre, Kobe 651-0073, Japan; kayanor@who.int; 8 School of Occupational Therapy, Social Work and Speech Pathology, Curtin University, Perth 6845, Australia; m.wright@curtin.edu.au; 9 Department of Physiology, Faculty of Medicine, General Sir John Kotelawala Defense University, Rathmalana 10390, Sri Lanka; lasakamd@ymail.com; 10 Hyogo Institute for Traumatic Stress, Kobe 651-0073, Japan; kato@j-hits.org (H.K.); osawa@j-hits.org (T.O.); 11 Child and Community Wellbeing Unit, Melbourne School of Population and Global Health, University of Melbourne, Melbourne 3010, Australia; lgibbs@unimelb.edu.au

**Keywords:** disaster, mental health, psychosocial, Asia Pacific, COVID-19, Health EDRM

## Abstract

Addressing the psychological mechanisms and structural inequalities that underpin mental health issues is critical to recovery following disasters and pandemics. The Asia Pacific Disaster Mental Health Network was established in June 2020 in response to the current disaster climate and to foster advancements in disaster-oriented mental health research, practice and policy across the region. Supported by the World Health Organization (WHO) Thematic Platform for Health Emergency and Disaster Risk Management (Health EDRM), the network brings together leading disaster psychiatry, psychology and public health experts. Our aim is to advance policy, research and targeted translation of the evidence so that communities are better informed in preparation and response to disasters, pandemics and mass trauma. The first meetings of the network resulted in the development of a regional disaster mental health agenda focused on the current context, with five priority areas: (1) Strengthening community engagement and the integration of diverse perspectives in planning, implementing and evaluating mental health and psychosocial response in disasters; (2) Supporting and assessing the capacity of mental health systems to respond to disasters; (3) Optimising emerging technologies in mental healthcare; (4) Understanding and responding appropriately to addressing the mental health impacts of climate change; (5) Prioritising mental health and psychosocial support for high-risk groups. Consideration of these priority areas in future research, practice and policy will support nuanced and effective psychosocial initiatives for disaster-affected populations within the Asia Pacific region.

## 1. Introduction

Disasters create an environment of disruption, trauma and grief, with potential for sustained mental health impacts. Heightened stress during pandemics and disasters can impair individual wellbeing (with effects on psychological health and sleep), cognitive function (memory, concentration and executive function), high-risk behaviours (alcohol and substance use, increased rates of domestic violence) and behavioural outcomes (such as compliance or disregard for public health orders) [1,2]. The mental health effects of disasters result not only from trauma exposure but may also arise from the implementation of public health response strategies such as quarantine, physical and social distancing or evacuation [3,4]. Economic insecurity, unemployment or underemployment, school closures and the shutdown of regional infrastructure can have devastating effects for population mental health. In addition, concerns regarding safety measures and sufficient supply of personal protective equipment can contribute to psychological distress [4,5]. Moreover, while many people demonstrate tremendous resilience during emergencies and in the immediate aftermath [6], the long-term psychological effects of disasters and pandemics are often debilitating [7,8].

The Asia Pacific region records the highest frequency of hazards and greatest number of people affected by disasters annually [9,10]. The immense psychological consequences are particularly concerning in nations with developing mental health systems and services [11]. A significant proportion of mental health need is unmet, and this has substantial effects on the social ecology and economic stability of affected communities. As climate change, growing urbanization, population density and animal–human viral transmission generate increasingly severe impacts of hazards and health emergencies, attention to mental health will be critical.

In response, the Asia Pacific Disaster Mental Health Network was established in June 2020 to create a collaborative platform for rigorous research, evidence-based practice and tailored policy designed to support improvements in mental health among disaster-affected communities. The network is supported by the World Health Organization (WHO) Thematic Platform for Health Emergency and Disaster Risk Management (Health EDRM) and its research network [12]. At its formation, the network represented seven Asian-Pacific nations, and it is growing. The network’s membership represents practitioners and scholars with broad interdisciplinary expertise in the fields of humanitarian response, psychiatry, psychology, public health, disaster risk reduction, human rights and security, indigenous mental health, emergency and mental health services and climate change action. In its first meetings, the network sought to determine an agenda for the advancement of disaster mental health evidence and practice within the region that would inform the design of future collaborative research, policy development and delivery of services.

## 2. Materials and Methods

The Asia Pacific Disaster Mental Health Network comprises fifteen representatives (57% female), selected for their expertise in responding to health emergencies and natural disasters, and work with trauma-affected communities within the region. The network is open to all Asia Pacific nations and currently includes representatives from Australia, Japan, China, Nepal, Sri Lanka, India and the USA. The purpose of the network’s early meetings was to establish a collaborative platform for future research and policy development and set overarching priorities in line with the goals of the WHO Thematic Platform for Health EDRM and its research network. Monthly meetings are conducted via videoconference. The selection of research priorities was conducted through open consultation within the network. An iterative-generative reflective method was adopted, whereby experiential knowledge of network members gleaned through their immersion with affected communities allowed for iterative debate [13]. As reflective-generative practitioners, each network member provided community-relevant insights as to potential priority areas. An iterative and deliberative discussion occurred across two meetings, held in June and July 2020. A list of key priorities identified by the group as central to mental health practice, research and policy within the region was generated. Thirteen representatives attended the first meeting and ten representatives attended the second. Impromptu responses on the priority list were systematically collated; the list was adapted following a second round of discussion and then circulated among the representatives for feedback. An additional round of input from representatives on the priorities and supporting evidence was incorporated into the write-up. All network members contributed to and provided feedback on the final priority list.

## 3. Results

### 3.1. Key Priorities for Disaster Mental Health

Large-scale climate disasters, severe wildfires and the COVID-19 pandemic have drawn renewed global attention to disaster response this year. The COVID-19 pandemic has amplified the structural injustices of race, faith, gender, age, migration and economic inequality within and across societies, with significant implications for mental health (e.g., [14,15,16,17,18]). Addressing structural disadvantage and inequality is vital, and without attention to these issues, many mental health difficulties during and after disasters may not be amenable to psychological treatment. Mental health practitioners and researchers play a vital role in highlighting injustice, community needs and the role of economic empowerment in supporting mental health. It is critical that psychological first aid and evidence-based interventions suitable for response to mass trauma events are implemented to support individual, family and community level improvements in mental health, recognizing that psychological distress occurs on a continuum and multi-level strategies are required [19,20]. First, broad public health strategies such as psychological first aid, mental health education, family reunification and child-friendly spaces, should be implemented at the community level to address general distress following an event; second, delivery of low-intensity programs to assist those dealing with sustained distress and psychological difficulties; third, clinical treatment provided for those with diagnosable conditions [19,21,22]. However, the effectiveness of treatments will vary due to the sociocultural context in which they are delivered [23]. A nuanced, solution-based approach will support significant advancements in this field. In line with this point, we identified the following priorities for a regional disaster mental health agenda.

### 3.2. Strengthening Community Engagement and the Integration of Diverse Perspectives in Planning, Implementing and Evaluating Mental Health Response

Reinforcing local social networks, social solidarity and engagement with community groups in responding to disasters and other mass trauma events will enhance psychosocial outcomes [24,25,26]. Community-driven responses are led by the community and may invite external agency partnership, whereas community-supported responses are facilitated by an external agency with the endorsement of the community [27]. Central to both approaches is that the community be recognised as valued authorities on their own lived experience. Listening to and incorporating diverse knowledges and multiple perspectives are essential to ensure that mental health services and psychosocial initiatives designed for any community are accessible, acceptable, culturally secure and developmentally appropriate [28] and that intervention models, disaster risk reduction strategies and mental health policy are designed and delivered in ways that are meaningful and relevant [29]. Furthermore, emerging evidence suggests that mutual reinforcement of public health messages and actions among community members has positive implications for health-related behaviors and compliance with public health directives during pandemics [30]. Restoring connections to the natural environment will have additional mental health benefits [31]. Working within existing community social structures and across a broad cross section of the community—with Elders, youth, local faith leaders and community groups—helps to establish respectful and collaborative relationships. Measures to access broad input and community guidance will result in treatment models, services and strategies that meet the diversity of mental health needs [32,33,34].

### 3.3. Supporting the Capacity of Mental Health Systems to Respond to Disasters

Disasters create multiple waves of healthcare need. Early response requires a focus on physical injuries, bereavement and re-establishing critical infrastructure for survival [35]. Establishing conditions for safe recovery will lessen distress for a vast majority of the population [19,36]. Mental illness tends to emerge in a second surge of health need, often months and years following the initial emergency [37]. However, the COVID-19 pandemic highlighted mental health needs that required immediate support during this crisis [2,5]. Health systems need to be prepared to address the short- and long-term mental health needs that arise within disaster-affected settings. Disaster response must begin with a diverse and well-supported workforce and include ensuring that workers are trained and supported with ongoing supervision and further training in psychological care for traumatized people—including how to cope with the added overlay of mass trauma impacts. Where disasters are more likely to affect remote areas, infrastructure to support regional health workers and digital health platforms will be important [38]. Lessons learned from the COVID-19 pandemic have already sparked significant expansion of mental health systems in many nations, including in China, where an increased workforce was engaged in order to address the psychological distress and grief arising from the pandemic in Wuhan [39,40]. Similar initiatives have been developed in other parts of the Asia Pacific region, including increased mental health budget funding in Australia [41], improved and expanded mental health helpline services in Nepal [42] and increased in-person and remote counselling services in Japan [43]. The challenge will be an ongoing commitment to the long-term sustainability of services to address the growing incidence of disaster-related trauma and grief within our region and ensuring that first responders, medical, nursing and allied health staff are well supported [44,45]. Mental health services research is both urgent and critical to evaluate the efficacy of models for rapid upskilling and ongoing support of the healthcare workforce, and to design and implement the appropriate expansion of access; acceptability and cultural security of services; effectiveness of trauma-informed treatments in low resource settings and community-based strategies to prevent the escalation of psychological distress.

### 3.4. Optimising the Integration of Digital Platforms in Mental Healthcare to Support Access and Acceptability of Care

The COVID-19 pandemic has fast-tracked the development and widespread adoption of technology in mental healthcare in many settings. Digital mental health services include treatment sessions conducted via video call, telephone helplines, clinical text messaging services, digital health applications and platforms, online streaming and therapy services and mass dissemination of mental health resources on social media [39,46]. Enabling the safe continuation of mental health services during lockdown or physical distancing, tele-mental health services have been widely implemented in many nations including China, Japan, Australia and New Zealand and have received additional funding for development and dissemination globally [39,47,48]. New and adapted technologies have the potential to transform the delivery of mental healthcare, as care can be tailored on a personal level, provided anywhere, be perceived as less stigmatizing and can empower people to take a more active role in their own healthcare decisions [49,50,51]. Although many settings within the Asia Pacific region still lack reliable access to electricity, internet and phone coverage, all limiting the use of digital mental healthcare [52]; the growing ubiquity of smartphone use has enabled rapid communication of disaster and mental health messaging, reaching populations less likely to be engaged with mainstream health services, such as international migrant workers [53]. Technology may also enable people to maintain the social connections that are critical to mental health and wellbeing outcomes. However, the digitalization of mental healthcare has possible negative implications. Reliance on technology can lead to social disengagement, and there are increasing concerns about the potential for misinformation with unregulated online information, as well as technical issues, unreliable internet access, low digital competences of health providers and clients, safety of data handling and perceived loss of therapeutic relationships [49,54]. It is thus essential to now identify which services and strategies have proven to be protective and efficient in improving mental health outcomes in the context of COVID-19 so as to design approaches that will have relevance beyond this pandemic.

### 3.5. Addressing the Mental Health Impacts of Climate Change

Climate change has increased the frequency and severity of natural disasters in the Asia Pacific region, with resulting risks for mental health problems [55,56,57]. The relationship between climate change and mental health impacts can be direct, by experiencing trauma caused by climate hazards, or indirect, through resulting physical health consequences, increased economic vulnerability and detrimental effects on community cohesion [55,56]. Further indirect effects from climate change may arise through a reduced sense of hope and self- and community-efficacy, identified as essential elements in recovery from mass trauma events such as natural disasters [58]. Within the Asia Pacific region, Indigenous communities and those dependent on agricultural production or coastal fishing experience disproportionate adverse impacts of climate change [55,56,59]. However, the effects are broadening—the Australian 2020 wildfires demonstrated widespread ecological and economic damage—with substantial psychological effects [38,60]. Similarly, climate change has had significant effects on mental health in the Pacific Island of Tuvalu [59], where the changing climate threatens irreversible changes to the way of life [61]. Climate-related hazards (i.e., tropical cyclones and increasing ocean temperatures) combined with urbanization, land shortages, overcrowding, limitations in infrastructure, services and poor governance have resulted in high levels of stress, anxiety and depressive symptoms among the Tuvaluan population [59]. Similar issues accompanied the impact of 2013 super typhoon Haiyan in the Philippines [62]. Direct and indirect mental health consequences from climate change are current and understudied [57]. This gap in our knowledge requires our immediate and collective attention in order to bring about an efficient, effective and holistic approach to mitigating the inequity of climate change impacts on mental health, led by local experts. This effort must become a central focus of disaster risk reduction in the coming decades.

### 3.6. Prioritising Mental Health and Psychosocial Support for High-Risk Groups

High-risk groups, including those disadvantaged or discriminated against due to the characteristics and intersection of age, gender, sexuality, ethnicity, faith, ability, migration and economic status, may be at greater risk of mental health difficulties during and after disasters [63,64]. In addition, those affected by domestic violence, chronic mental illness, forced displacement, job loss or homelessness will require tailored solutions [65]. The damage to the natural environment from climatic hazards may also generate an additional level of pain and loss for First Nations people with historical and cultural connections to the land [66], as well as for many others who find solace and peace in the persistence of nature. Failure to recognize historical circumstances and cultural values can result in interventions reinforcing existing patterns of disadvantage and prejudice [67]. Risk factors are dynamic, and an individual’s level of vulnerability during disasters is dependent on a range of contextual factors, resulting in resilience at times and vulnerability at others [63]. For example, there is a complex relationship between disaster exposure and suicide risk, with increased risk associated with large-scale disaster impacts and length of time following exposure [68]. Specific groups, such as working-age men and older women, and factors including limited social connections, economic insecurity, living in temporary housing and pre-existing or new mental health conditions may increase suicide risk following disasters [68,69]. COVID-19 has demonstrated the potential for mental health risks to emerge as a result of both disaster-related trauma and the public health safety measures implemented to reduce transmission. This has been highlighted in Nepal, where widespread job loss and economic insecurity arising from government lockdown measures during the pandemic has resulted in a tragic spike in suicides, with 1200 deaths reported due to suicide during the 74 day lockdown [70,71]. Established mental health services in Nepal are working to provide helpline services through telephone and social media and further improve the capacity of community psychosocial workers to respond to individuals experiencing psychological distress [42]. Thus, effective services working within high-risk communities must be supported to continue and, where needed, expand their services during and after disasters. As we see an increasingly sophisticated global response to disaster risk reduction, inclusion and support for high-risk groups will be vital for effective mental healthcare.

## 4. Conclusions

The Asia Pacific Disaster Mental Health Network was established to foster advancements and coordination of psychosocial supports and mental health service delivery, policy development and collaborative research in the region. In line with the priorities of the WHO Thematic Platform for Health EDRM and its research network [12,72], the Asia Pacific Network aims to contribute to improvements in mental healthcare and psychosocial support through rigorous research and policy. Within the context of the COVID-19 pandemic and recent climatic hazards, the network set an agenda that prioritises strengthened community engagement, improved capacity for mental health and community services to respond to the needs of disaster-affected populations, integrating emerging technologies, addressing the impacts of climate change and supporting high-risk groups. Through multidisciplinary regional partnerships, the network will contribute to effective and culturally secure intervention design and delivery, translation of evidence to support community preparedness and response, and the collection of high-quality data to inform knowledge, policy and practice specific to the Asia Pacific region and relevant across the globe.

## References

[1] Holmes E.A., O’Connor R.C., Perry V.H., Tracey I., Wessely S., Arseneault L., Ballard C., Christensen H., Silver R.C., Everall I. (2020). Multidisciplinary research priorities for the COVID-19 pandemic: A call for action for mental health science. Lancet Psychiatry.

[2] Pfefferbaum B., North C.S. (2020). Mental health and the Covid-19 pandemic. N. Engl. J. Med..

[3] Brooks S.K., Webster R.K., Smith L.E., Woodland L., Wessely S., Greenberg N., Rubin G.J. (2020). The psychological impact of quarantine and how to reduce it: Rapid review of the evidence. Lancet.

[4] Li H.Y., Cao H., Leung D.Y., Mak Y.W. (2020). The psychological impacts of a COVID-19 outbreak on college students in China: A longitudinal study. Int. J. Environ. Res. Public Health.

[5] Choi E.P.H., Hui B.P.H., Wan E.Y.F. (2020). Depression and anxiety in Hong Kong during COVID-19. Int. J. Environ. Res. Public Health.

[6] Bonanno G.A., Brewin C.R., Kaniasty K., Greca A.M.L. (2010). Weighing the costs of disaster: Consequences, risks, and resilience in individuals, families, and communities. Psychol. Sci. Public Interest.

[7] Beaglehole B., Mulder R.T., Frampton C.M., Boden J.M., Newton-Howes G., Bell C.J. (2018). Psychological distress and psychiatric disorder after natural disasters: Systematic review and meta-analysis. Br. J. Psychiatry.

[8] Bryant R.A., Gibbs L., Gallagher H.C., Pattison P., Lusher D., MacDougall C., Harms L., Block K., Sinnott V., Ireton G. (2018). Longitudinal study of changing psychological outcomes following the Victorian black Saturday bushfires. Aust. N. Z. J. Psychiatry.

[9] CRED, UNISDR (2019). 2018 Review of Disaster Events.

[10] Below R., Wallemacq P. (2018). Annual Disaster Statistical Review 2017.

[11] Henderson L.J. (2004). Emergency and disaster: Pervasive risk and public bureaucracy in developing nations. Public Organ. Rev..

[12] Kayano R., Chan E.Y., Murray V., Abrahams J., Barber S.L. (2019). WHO Thematic Platform for Health Emergency and Disaster Risk Management Research Network (TPRN): Report of the Kobe Expert Meeting. Int. J. Environ. Res. Public Health.

[13] Dokecki P.R. (1992). On knowing the community of caring persons: A methodological basis for the reflective-generative practice of community psychology. J. Community Psychol..

[14] Matache M., Bhabha J. (2020). Anti-Roma Racism is Spiraling during COVID-19 Pandemic. Health Hum. Rights.

[15] Tai D.B.G., Shah A., Doubeni C.A., Sia I.G., Wieland M.L. (2020). The Disproportionate Impact of COVID-19 on Racial and Ethnic Minorities in the United States. Clin. Infect. Dis..

[16] Kihato C.W., Landau L.B. (2020). Coercion or the social contract? COVID 19 and spatial (in) justice in African cities. City Soc..

[17] Xafis V. (2020). ‘What is Inconvenient for You is Life-saving for Me’: How Health Inequities are playing out during the COVID-19 Pandemic. Asian Bioeth. Rev..

[18] Pirtle W.N.L. (2020). Racial capitalism: A fundamental cause of novel coronavirus (COVID-19) pandemic inequities in the United States. Health Educ. Behav..

[19] Tol W.A., Barbui C., Galappatti A., Silove D., Betancourt T.S., Souza R., Golaz A., Van Ommeren M. (2011). Mental health and psychosocial support in humanitarian settings: Linking practice and research. Lancet.

[20] Zhang Y., Ma Z.F. (2020). Impact of the COVID-19 pandemic on mental health and quality of life among local residents in Liaoning Province, China: A cross-sectional study. Int. J. Environ. Res. Public Health.

[21] Forbes D., O’Donnell M., Bryant R. (2017). Psychosocial recovery following community disasters: An international collaboration. Aust. N. Z. J. Psychiatry.

[22] Kim Y., Akiyama T. (2011). Post-disaster mental health care in Japan. Lancet.

[23] Newnham E.A., McBain R.K., Hann K., Akinsulure-Smith A.M., Weisz J., Lilienthal G.M., Hansen N., Betancourt T.S. (2015). The Youth Readiness Intervention for war-affected youth. J. Adolesc. Health.

[24] Bryant R., Gallagher H., Waters E., Gibbs L., Pattison P., MacDougall C., Harms L., Block K., Baker E., Sinnott V. (2017). Mental health and social networks after disaster. Am. J. Psychiatry.

[25] Gallagher H.C., Block K., Gibbs L., Forbes D., Lusher D., Molyneaux R., Richardson J., Pattison P., MacDougall C., Bryant R.A. (2019). The effect of group involvement on post-disaster mental health: A longitudinal multilevel analysis. Soc. Sci. Med..

[26] Hawdon J., Räsänen P., Oksanen A., Ryan J. (2012). Social solidarity and wellbing after critical incidents: Three cases of mass shootings. J. Crit. Incid. Anal..

[27] Rodriguez-García R., Bonnel R., N’Jie N.D., Olivier J., Pascual F.B., Wodon Q. (2011). Analyzing Community Responses to HIV and AIDS: Operational Framework and Typology.

[28] Wright M., Lin A., O’Connell M. (2016). Humility, inquisitiveness, and openness: Key attributes for meaningful engagement with Nyoongar people. Adv. Ment. Health.

[29] Tol W.A., Song S., Jordans M.J. (2013). Annual research review: Resilience and mental health in children and adolescents living in areas of armed conflict–a systematic review of findings in low-and middle-income countries. J. Child Psychol. Psychiatry.

[30] Lim S., Nakazato H. (2020). The emergence of risk communication networks and the development of citizen health-related behaviors during the COVID-19 pandemic: Social selection and contagion processes. Int. J. Environ. Res. Public Health.

[31] Block K., Molyneaux R., Gibbs L., Alkemade N., Baker E., MacDougall C., Ireton G., Forbes D. (2019). The role of the natural environment in disaster recovery: “We live here because we love the bush”. Health Place.

[32] Harr C.R., Yancey G.I. (2014). Social work collaboration with faith leaders and faith groups serving families in rural areas. J. Relig. Spiritual. Soc. Work Soc. Thought.

[33] Israel B.A., Schulz A.J., Parker E.A., Becker A.B. (1998). Review of community-based research: Assessing partnership approaches to improve public health. Annu. Rev. Public Health.

[34] Wright M., Crisp N., Newnham E.A., Flavell H., Lin A. (2020). Addressing mental health in Aboriginal young people in Australia. Lancet Psychiatry.

[35] Bar-On E., Abargel A., Peleg K., Kreiss Y. (2013). Coping with the challenges of early disaster response: 24 years of field hospital experience after earthquakes. Disaster Med. Public Health Prep..

[36] North C.S., Pfefferbaum B. (2013). Mental health response to community disasters: A systematic review. JAMA.

[37] Gibbs L., Waters E., Bryant R.A., Pattison P., Lusher D., Harms L., Richardson J., MacDougall C., Block K., Snowdon E. (2013). Beyond Bushfires: Community, Resilience and Recovery-a longitudinal mixed method study of the medium to long term impacts of bushfires on mental health and social connectedness. BMC Public Health.

[38] Newnham E.A., Titov N., McEvoy P. (2020). Preparing mental health systems for climate crisis. Lancet Planet. Health.

[39] Liu S., Yang L., Zhang C., Xiang Y.T., Liu Z., Hu S., Zhang B. (2020). Online mental health services in China during the COVID-19 outbreak. Lancet Psychiatry.

[40] Li W., Yang Y., Liu Z.H., Zhao Y.J., Zhang Q., Zhang L., Cheung T., Xiang Y.T. (2020). Progression of mental health services during the COVID-19 outbreak in China. Int. J. Biol. Sci..

[41] Australian Government, Department of Health (2020). Supporting the Mental Health of Australians through the Coronavirus Pandemic.

[42] MHIN A global Community of Mental Health Innovators, and Department of Mental Health and Substance Use WHO. Resources for Mental Health and Psychosocial Support during the COVID-19 Pandemic. https://www.mhinnovation.net/collaborations/resources-mental-health-and-psychosocial-support-during-covid-19-pandemic.

[43] Health, Centre for Japanese Mental In-Person and Remote Counseling. https://cjmh.org/counseling/.

[44] Matsuishi K., Kawazoe A., Imai H., Ito A., Mouri K., Kitamura N., Miyake K., Mino K., Isobe M., Takamiya S. (2012). Psychological impact of the pandemic (H1N1) 2009 on general hospital workers in Kobe. Psychiatry Clin. Neurosci..

[45] Krystal J.H., McNeil R.L. (2020). Responding to the hidden pandemic for healthcare workers: Stress. Nat. Med..

[46] Ben-Zeev D. (2020). The digital mental health genie is out of the bottle. Psychiatr. Serv..

[47] Zhou X., Snoswell C.L., Harding L.E., Bambling M., Edirippulige S., Bai X., Smith A.C. (2020). The role of telehealth in reducing the mental health burden from COVID-19. Telemed. E-Health.

[48] Marshall J.M., Dunstan D.A., Bartik W. (2020). The role of digital mental health resources to treat trauma symptoms in australia during COVID-19. Psychol. Trauma Theory Res. Pract. Policy.

[49] Bucci S., Schwannauer M., Berry N. (2019). The digital revolution and its impact on mental health care. Psychol. Psychother. Theory Res. Pract..

[50] Hollis C., Sampson S., Simons L., Davies E.B., Churchill R., Betton V., Butler D., Chapman K., Easton K., Gronlund T.A. (2018). Identifying research priorities for digital technology in mental health care: Results of the James Lind Alliance Priority Setting Partnership. Lancet Psychiatry.

[51] World Economic Forum (2019). Empowering 8 Billion Minds: Enabling Better Mental Health for All Via the Ethical Adoption of Technologies.

[52] Statica Internet Penetration Rate in Asia Compared to the Global Penetration Rate from 2009 to 2020. https://www.statista.com/statistics/265156/internet-penetration-rate-in-asia/.

[53] Liem A., Wang C., Wariyanti Y., Latkin C.A., Hall B.J. (2020). The neglected health of international migrant workers in the COVID-19 epidemic. Lancet Psychiatry.

[54] Christensen H., Griffiths K.M., Farrer L. (2009). Adherence in internet interventions for anxiety and depression: Systematic review. J. Med. Internet Res..

[55] Berry H.L., Bowen K., Kjellstrom T. (2010). Climate change and mental health: A causal pathways framework. Int. J. Public Health.

[56] Fritze J.G., Blashki G.A., Burke S., Wiseman J. (2008). Hope, despair and transformation: Climate change and the promotion of mental health and wellbeing. Int. J. Ment. Health Syst..

[57] Hayes K., Blashki G., Wiseman J., Burke S., Reifels L. (2018). Climate change and mental health: Risks, impacts and priority actions. Int. J. Ment. Health Syst..

[58] Hobfoll S.E., Watson P., Bell C.C., Bryant R.A., Brymer M.J., Friedman M.J., Friedman M., Gersons B.P., De Jong J.T., Layne C.M. (2007). Five essential elements of immediate and mid–term mass trauma intervention: Empirical evidence. Psychiatrynterpers. Biol. Process..

[59] Gibson K., Haslam N., Kaplan I. (2019). Distressing encounters in the context of climate change: Idioms of distress, determinants, and responses to distress in Tuvalu. Transcult. Psychiatry.

[60] Miller V.M., Davies M.J., Etherton-Beer C., McGough S., Schofield D., Jensen J.F., Watson N. (2020). Increasing patient activation through diabetes self-management education: Outcomes of DESMOND in regional Western Australia. Patient Educ. Couns..

[61] Nunn P.D. (2013). The end of the P acific? Effects of sea level rise on P acific I sland livelihoods. Singap. J. Trop. Geogr..

[62] Chan C., Tang K., Hall B., Yip S., Maggay M. (2016). Psychological sequelae of the 2013 Super Typhoon Haiyan among survivor-responders. Psychiatry Interpers. Biol. Process..

[63] Newnham E.A., Ho J.Y., Chan E.Y.Y., Clarke M., Murray V., Chan E.Y.Y., Kayano R., Abrahams J., O’Sullivan T. (2020). Identifying and engaging high-risk groups in disaster research. World Health Organization Guidance on Research Methods for Health Emergency and Disaster Risk Management.

[64] Collins P.H., Bilge S. (2016). Intersectionality.

[65] Chan E.Y.Y., Gobat N., Kim J.H., Newnham E.A., Huang Z., Hung H., Dubois C., Hung K.K.C., Wong E.L.Y., Wong S.Y.S. (2020). Informal home care providers: The forgotten health-care workers during the COVID-19 pandemic. Lancet.

[66] Williamson B., Markham F., Weir J. (2020). Aboriginal Peoples and the Response to the 2019−2020 Bushfires.

[67] Hsu M., Howitt R., Miller F. (2015). Procedural Vulnerability and Institutional Capacity Deficits in Post-Disaster Recovery and Reconstruction: Insights from Wutai Rukai Experiences of Typhoon Morakot. Hum. Organ..

[68] Matsubayashi T., Sawada Y., Ueda M. (2013). Natural disasters and suicide: Evidence from Japan. Soc. Sci. Med..

[69] Safarpour H., Sohrabizadeh S., Malekyan L., Safi-Keykaleh M., Pirani D., Daliri S., Bazyar J. (2020). Suicide death rate after disasters: A meta-analysis study. Arch. Suicide Res..

[70] Singh R., Baral K.P., Mahato S. (2020). An urgent call for measures to fight against increasing suicides during COVID-19 pandemic in Nepal. Asian J. Psychiatry.

[71] Poudel A. (2020). Over 1,200 people killed themselves during 74 days of lockdown in Nepal. The Kathmandu Post.

[72] Newnham E.A., Reifels L., Gibbs L., larke M., Murray V., Chan E.Y.Y., Kayano R., Abrahams J., O’ Sullivan T. (2020). Disaster mental health research. World Health Organization Guidance on Research Methods for Health Emergency and Disaster Risk Management.

